# Acquired Fanconi syndrome in patients with *Legionella* pneumonia

**DOI:** 10.1186/1471-2369-14-171

**Published:** 2013-08-02

**Authors:** Naoko Kinoshita-Katahashi, Hirotaka Fukasawa, Sayaka Ishigaki, Shinsuke Isobe, Shiro Imokawa, Yoshihide Fujigaki, Ryuichi Furuya

**Affiliations:** 1 Renal Division, Department of Internal Medicine, Iwata City Hospital, 512-3 Ohkubo, Iwata, Shizuoka, 438–8550, Japan; 2 Internal Medicine1, Hamamatsu University School of Medicine, 1-20-1 Handayama, Higashi-ku, Hamamatsu, Shizuoka 431-3192, Japan; 3 Department of Respiratory Medicine, Iwata City Hospital, 512-3 Ohkubo, Iwata, Shizuoka 438-8550, Japan

**Keywords:** Fanconi syndrome, *Legionella* pneumonia, Electrolyte abnormality

## Abstract

**Background:**

Hyponatremia is often observed in patients with *Legionella* pneumonia. However, other electrolyte abnormalities are uncommon and the mechanism remains to be clarified.

**Case presentation:**

We experienced two male cases of acquired Fanconi syndrome associated with *Legionella* pneumonia. The laboratory findings at admission showed hypophosphatemia, hypokalemia, hypouricemia and/or hyponatremia. In addition, they had the generalized dysfunction of the renal proximal tubules presenting decreased tubular reabsorption of phosphate (%TRP), increased fractional excretion of potassium (FEK) and uric acid (FEUA), low-molecular-weight proteinuria, panaminoaciduria and glycosuria. Therefore, they were diagnosed as Fanconi syndrome. Treatment for *Legionella* pneumonia with antibiotics resulted in the improvement of all serum electrolyte abnormalities and normalization of the %TRP, FEK, FEUA, low-molecular-weight proteinuria, panaminoaciduria and glycosuria, suggesting that *Legionella pneumophila* infection contributed to the pathophysiology of Fanconi syndrome.

**Conclusion:**

To the best of our knowledge, this is the first report demonstrating Fanconi syndrome associated with *Legionella* pneumonia.

## Background

Fanconi syndrome is a generalized dysfunction of the renal proximal tubules without primary glomerular involvement. It is typically characterized by variable degrees of phosphate, amino acids or glucose wasting by the proximal tubules. In addition, hypokalemia, hypouricemia, metabolic acidosis and low-molecular-weight proteinuria can be part of the clinical spectrum [1]. Acquired forms of Fanconi syndrome are caused by paraproteinemia, Sjögren syndrome, primary biliary cirrhosis (PBC) and drugs such as cisplatin [2-4]. However, infectious diseases have not been reported as a cause of Fanconi syndrome.

In this report, we present extremely rare cases of *Legionella* pneumonia complicated by Fanconi syndrome.

## Case presentation

### Patient 1

A 75-year-old man was admitted to our hospital with fever and general malaise.

A physical examination at the time of admission showed a body temperature of 38.9°C, blood pressure of 120/70 mmHg and a pulse rate of 80 beats/min. Laboratory findings showed a white blood cell count of 13,400/mm^3^ and C-reactive protein (CRP) of 23.7 mg/dL. A chest computed tomography (CT)-scan showed a ground-glass appearance in the right middle and lower lobes. Because the urinary antigen test for *Legionella pneumophila* serotype 1 (BinaxNOW® *Legionella*, Binax, Inc. ME, USA) was positive, he was diagnosed as *Legionella* pneumonia.

Several electrolyte abnormalities coexisted including hyponatremia, hypokalemia, hypophosphatemia and hypouricemia. His renal function was normal and the arterial blood gases analysis showed mild metabolic acidosis (Table 1). The tubular reabsorption of phosphate (%TRP) was decreased, although the fractional excretion of uric acid (FEUA) was increased, indicating disturbed proximal tubular reabsorption. Despite the hypokalemia, his urinary potassium excretion was not suppressed. Elevations in urinary levels of β2-microglobulin (β2-MG) and N-acetyl-β-D-glucosaminidase (NAG), glycosuria and panaminoaciduria were also observed (Tables 1 and 2). Therefore, he was diagnosed as Fanconi syndrome.

**Table 1:** Laboratory data on admission

	**Normal range**	**Patient 1**	**Patient 2**
BUN	8–22 mg/dL	17	41
Serum creatinine	0.71–1.20 mg/dL	0.88	2.23
Serum sodium	136–147 mEq/L	121	143
Serum potassium	3.6–5.0 mEq/L	3.4	3.5
Serum phosphorus	2.5–4.3 mg/dL	1.3	2.0
Serum uric acid	< 7.0 mg/dL	2.4	2.7
Serum chloride	101–109 mEq/L	83	107
Serum CPK	56–244 IU/L	670	2,330
Bicarbonate	22.0–26.0 mEq/L	20.8	15.7
Anion gap	12 ± 2 mEq/L	15.2	20.3
Serum osmolarity	285–295 mOsm/kg	255	N.D.
Urinary osmolarity	mOsm/kg	673	N.D.
ADH	0.3–4.2 pg/mL	1.7	N.D.
Proteinuria	Negative; (−)	(2+)	(2+)
Leukocyturia	Negative; (−)	(−)	(−)
Urinary pH	4.8–7.5	6.5	5.5
Urinary glucose	Negative; (−)	(3+)	(2+)
Urinary RBC	0–4/HPF	10–19/HPF	10–19/HPF
%TRP	81–90%	54.0	51.0
FEK	10.0–20.0%	18.4	33.1
FEUA	5.5–11.0%	18.7	42.8
β2-MG	< 250 μg/L	80,397	110,556
NAG	< 7.0 U/L	18.0	19.7

Definition of the abbreviations: *ADH* antidiuretic hormone, *BUN* blood urea nitrogen, *CPK* creatine phosphokinase, *FEK* fractional excretion of potassium, *FEUA* fractional excretion of uric acid, *HPF* high power field, *NAG* N-acetyl-β-D-glucosaminidase, *N.D.* not determined, *RBC* red blood cell, *%TRP* tubular reabsorption of phosphate, *WBC* white blood cell, *β2-MG* β2-microglobulin. Conversion factors for units: BUN in mg/dL to mmol/L, × 0.357, creatinine in mg/dL to mmol/L, × 88.4, phosphorus in mg/dL to mmol/L, × 0.3229, uric acid in mg/dL to μmol/L, × 59.48, and ADH in pg/mL to pmol/L, × 0.923. No conversion is necessary for sodium, potassium, chloride, CPK, bicarbonate, anion gap, and osmolarity.

**Table 2:** Data of the analysis of urinary amino acids on admission

	**Normal range (mmol/day)**	**Patient 1**	**Patient 2**
Taurine	322.2–5214.5	1318.6	272.5
Phosphoethanolamine	31.0–110.0	49	34.4
Urea	130.3–493.2	223.9	341.3
Aspartic acid	< 12.7	N.D.	6.1
Hydroxyproline	N.D.	N.D.	N.D.
Threonine	79.9–528.3	1530.2	380.7
Serine	208.8–1020.0	1529.6	478.3
Asparagine	60.7–372.3	747.4	393.6
Glutamic acid	11.3–42.7	30.1	15.5
Glutamine	207.0–1357.3	1071.2	170.3
Sarcosine	< 99.0	24.8	N.D.
α-Aminoadipic acid	16.7–118.6	52.4	34.4
Proline	N.D.	18.4	25.6
Glycine	652.1–3670.6	852.4	336.2
Alanine	141.2–833.9	839.3	538.6
Citrulline	13.5–55.6	63.4	N.D.
α-Aminobutylic acid	< 27.1	N.D.	N.D.
Valine	24.8–82.2	97	104.2
Cystine	23.7–170.9	226.2	101.3
Cystathionine	< 44.7	21	334.6
Methionine	< 20.2	11.5	15.5
Isoleucine	7.5–23.5	26.5	29.2
Leucine	24.6–89.3	95.5	96.3
Tyrosine	50.6–308.4	327.4	111.8
Phenylalanine	27.2–110.2	150.7	199.8
γ-Amino β-hydroxybutyric acid	N.D.	N.D.	N.D.
β-Alanine	< 153.0	58.4	9.4
β-Amino-iso-butyric acid	< 1623.9	151.8	288.5
γ-Aminobutyric acid	N.D.	N.D.	N.D.
Monoethanolamine	195.3–606.2	292.8	356.8
Homocystine	N.D.	N.D.	N.D.
Histidine	436.4–2786.5	1392	496.8
3-Methylhistidine	113.4–480.9	202.9	396
1-Methylhistidine	59.3–2816.2	821.2	16.7
Carnosine	< 87.6	N.D.	21.2
Anserine	< 231.4	49.4	N.D.
Tryptophan	20.7–150.7	156.7	59.2
Hydroxylysine	< 22.9	3.8	N.D.
Ornithine	6.9–43.9	46.3	151.4
Lysine	51.6–1639.6	794.9	148.1
Arginine	11.6–54.8	51.5	31.3

Underlining indicates an abnormal value.

N.D. means not detected.

In addition, his urinary osmolarity was greater than his serum osmolarity and the level of antidiuretic hormone (ADH) was improperly high for the indicated serum osmolarity. There was no sign of extracellular fluid volume depletion. Therefore, a diagnosis of the syndrome of inappropriate secretion of ADH (SIADH) was also made.

**Figure 1:**
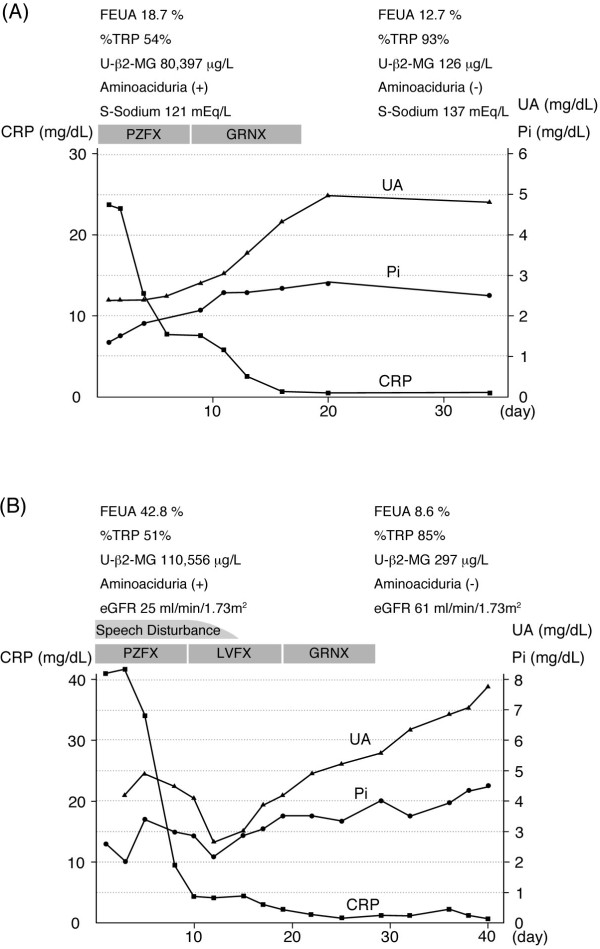
**Clinical course in Patient 1 (A) and Patient 2 (B).** Definition of the abbreviations: CRP = C-reactive protein, eGFR = estimated glomerular filtration rate, FEUA = fractional excretion of uric acid, GRNX = garenoxacin mesilate hydrate, LVFX = levofloxacin hydrate, Pi = phosphate (inorganic), PZFX = pazufloxacin mesilate, S-Sodium = serum sodium, UA = uric acid, U-β2-MG = urinary β2-microglobulin, %TRP = tubular reabsorption of phosphate.

He was treated for *Legionella* pneumonia with pazufloxacin mesilate (PZFX) at 1,000 mg per day. After treatment, his physical status recovered and his electrolyte abnormalities improved. Furthermore, his %TRP, FEUA, urinary β2-MG and NAG normalized, and the panaminoaciduria became undetectable (Figure 1A).

### Patient 2

A 57-year-old man was admitted to our hospital with fever and dysarthria.

A physical examination showed a body temperature of 39.1°C, a blood pressure of 112/48 mmHg and a pulse rate of 98 beats/min. Laboratory findings showed a white blood cell count of 6,100/mm^3^ and CRP of 46.7 mg/dL. A chest CT scan showed a ground-glass appearance in the left upper and lower lobes. Because of the positive urinary antigen test, he was diagnosed as *Legionella* pneumonia.

Several electrolyte abnormalities coexisted including hypophosphatemia, hypokalemia and mild hypouricemia. The arterial blood gas analysis showed metabolic acidosis (Table 1). The %TRP was decreased, although his fractional excretion of potassium (FEK) and FEUA were increased. Furthermore, his urinary β2-MG and NAG were remarkably increased (Table 1). Glycosuria and aminoaciduria were also observed (Table 2). Therefore, a diagnosis of Fanconi syndrome was made. Although his renal function was deteriorated, post-renal obstructive nephropathy and congenital anomalies were ruled out by the finding of abdominal CT scan.

Treatment for *Legionella* pneumonia was started with PZFX at 1,000 mg per day. After treatment, his symptoms disappeared and his electrolyte abnormalities improved. In addition, his %TRP, FEK, FEUA, urinary β2-MG and NAG normalized, and the aminoaciduria became undetectable (Figure 1B).

## Conclusions

Because the electrolyte abnormalities disappeared after treatment for *Legionella* pneumonia and there was no evidence of other causes of Fanconi syndrome, it is most likely that Fanconi syndrome was associated with *Legionella* pneumonia in our cases. This is the first report of acquired Fanconi syndrome caused by infectious diseases.

Hyponatremia is known as a relatively common electrolyte abnormality in patients with *Legionella* pneumonia [5]. The responsible mechanism of hyponatremia is thought to be SIADH. In accordance with this mechanism, the hyponatremia in Patient 1 was accompanied by improperly high levels of ADH and improved following the recovery from *Legionella* pneumonia.

However, other electrolyte abnormalities are uncommon in patients with *Legionella* pneumonia, and the precise mechanism remains to be clarified [5]. In our cases, the laboratory findings at admission also showed hypophosphatemia, hypokalemia and hypouricemia. In addition, remarkably increased urinary excretion of low-molecular-weight proteins such as β2-MG, decreased %TRP, increased FEK and FEUA, panaminoaciduria and glycosuria were coexisted. Therefore, our cases suggest that the proximal tubular dysfunction caused these serum electrolyte abnormalities.

A relationship has recently been proposed between mitochondrial disorders and Fanconi syndrome [6,7]. For instance, it is known that patients with mitochondrial disorders, including mitochondrial encephalomyopathy, lactic acidosis, and stroke-like episodes (MELAS), Kearns-Sayre syndrome, and anti-mitochondrial M2 antibody-positive primary biliary cirrhosis (PBC), often complicate with Fanconi syndrome [4,8,9]. Moreover, Fanconi syndrome related to antiviral agents, such as cidofovir, is thought to be caused by mitochondrial toxicity [10]. Because the mitochondria play a critical role in generating adenosine triphosphate (ATP) through oxidative phosphorylation and tubular epithelial cells, particularly in the renal proximal tubules, require a high energy supply from the mitochondria, it is reasonable that mitochondrial disorders can cause Fanconi syndrome. Interestingly, Evans *et al.* have reported that *Legionella pneumophila*, which is a facultative intracellular bacterium, was found inside renal tubular cells in autopsy cases of *Legionella* pneumonia [11]. In addition, it has been reported that *Legionella pneumophila* infection affects mitochondrial functions in mammalian cells [12,13]. Therefore, *Legionella pneumophila* infected the proximal tubular cells might interfere with their mitochondria and finally cause Fanconi syndrome in our cases.

However, we cannot explain the following issues: (i) from where *Legionella pneumophila* entered inside the renal proximal tubular cells (from basolateral or apical) and (ii) why the reabsorption defects developed only in the proximal tubules, not in the distal tubules. Furthermore, we cannot rule out the possibility that acute tubulointerstitial nephritis was occurred in the setting of *Legionella* pneumonia [14], although this possibility should be low because of the absence of typical symptoms such as rash and leukocyturia in our cases.

In summary, we experienced two rare cases of *Legionella* pneumonia complicated with Fanconi syndrome. Further studies are required to clarify the precise mechanisms responsible for the association between *Legionella pneumophila* infection and Fanconi syndrome.

## Consent

Written informed consents were obtained from the patients for publication of this Case report and any accompanying images. Copies of the written consents are available for review by the Series Editor of this journal.

## Abbreviations

ADH: Antidiuretic hormone; ATP: Adenosine triphosphate; BUN: Blood urea nitrogen; CRP: C-reactive protein; CT: Computed tomography; FEK: Fractional excretion of potassium; FEUA: Fractional excretion of uric acid; GRNX: Garenoxacin mesilate hydrate; LVFX: Levofloxacin hydrate; MELAS: Mitochondrial encephalomyopathy, lactic acidosis, and stroke-like episodes; NAG: N-acetyl-β-D-glucosaminidase; PBC: Primary biliary cirrhosis; PZFX: Pazufloxacin mesilate; SIADH: Syndrome of inappropriate ADH secretion; S-Sodium: Serum sodium; %TRP: Tubular reabsorption of phosphate; UA: Uric acid; β2-MG: β2-microglobulin.

## Competing interests

The authors declare that they have no competing interests.

## Authors’ contributions

NK, SaI, ShinI and ShirI treated the patient. NK wrote the first draft, and also evaluated the data. HF, RF and YF wrote the final draft. All authors reviewed and approved the final version of this manuscript.

## Pre-publication history

The pre-publication history for this paper can be accessed here:

http://www.biomedcentral.com/1471-2369/14/171/prepub
